# Identification and Characterization of Polymorphisms in piRNA Regions

**DOI:** 10.3390/cimb44020062

**Published:** 2022-02-15

**Authors:** José Roberto Sobrinho Lima, Jhully Azevedo-Pinheiro, Roberta Borges Andrade, André Salim Khayat, Paulo Pimentel de Assumpção, Ândrea Ribeiro-dos-Santos, Sidney Emanuel Batista dos Santos, Fabiano Cordeiro Moreira

**Affiliations:** 1 Núcleo de Pesquisas em Oncologia (NPO), Programa de Pós-Graduação em Oncologia e Ciências Médicas, Universidade Federal do Pará, Belém 66073-005, PA, Brazil; jsroberto.slima@gmail.com (J.R.S.L.); robertaborgesandrade@gmail.com (R.B.A.); khayatas@gmail.com (A.S.K.); assumpcaopp@gmail.com (P.P.d.A.); akely@ufpa.br (Â.R.-d.-S.); sidneysantosufpa@gmail.com (S.E.B.d.S.); 2 Laboratório de Genética Humana e Médica (LGHM), Programa de Pós-Graduação em Genética e Biologia Molecular, Universidade Federal do Pará, Belém 66075-110, PA, Brazil; jhully.pinheiro@icb.ufpa.br

**Keywords:** piRNA, polymorphisms, conservation

## Abstract

piRNAs are a class of noncoding RNAs that perform functions in epigenetic regulation and silencing of transposable elements, a mechanism conserved among most mammals. At present, there are more than 30,000 known piRNAs in humans, of which more than 80% are derived from intergenic regions, and approximately 20% are derived from the introns and exons of pre-mRNAs. It was observed that the expression of the piRNA profile is specific in several organs, suggesting that they play functional roles in different tissues. In addition, some studies suggest that changes in regions that encode piRNAs may have an impact on their function. To evaluate the conservation of these regions and explore the existence of a seed region, SNP and INDEL variant rates were investigated in several genomic regions and compared to piRNA region variant rates. Thus, data analysis, data collection, cleaning, treatment, and exploration were implemented using the R programming language with the help of the RStudio platform. We found that piRNA regions are highly conserved after considering INDELs and do not seem to present an identifiable seed region after considering SNPs and INDEL variants. These findings may contribute to future studies attempting to determine how polymorphisms in piRNA regions can impact diseases.

## 1. Introduction

The Encyclopedia of DNA Elements (ENCODE), a large consortium project to map all functional elements in the human genome, has suggested that up to 80% of the genome is biologically active and functional with an essential role in controlling DNA expression and spatial organization of the genome [1,2,3]. This regulation is carried out by DNA sequences that are transcribed into noncoding RNA molecules [2]. Three major families of small noncoding RNAs (sncRNAs) in eukaryotic cells have been widely studied: microRNAs (miRNAs), interference RNAs (siRNAs), and PIWI-interacting RNAs (piRNAs) [4].

piRNAs are a class of recently discovered sncRNAs that were described for the first time in germ cells [5,6,7,8] and identified later in somatic cells [9]. These sncRNAs have 24–31 nucleotides, interact with argonaut proteins of the PIWI subfamily, and form the PIWI–piRNA pathway, which plays roles in transcriptional and posttranscriptional silencing of transposable elements (TEs), epigenetic regulation, the maintenance of germ cell function, and the regulation of mRNA [9,10,11]. However, the most well-characterized piRNA function is TE silencing [11]. The silencing of TEs and other genetic elements in germlines, at both the transcriptional and the posttranscriptional levels, is highly conserved across animal species [12,13].

There are over 30,000 piRNAs in humans, among which more than 80% are derived from intergenic regions, and approximately 20% are derived from introns and exons of pre-mRNAs [14,15].

These sncRNAs, like miRNAs, can act by inducing mRNA repression through imperfect base pairing [12,16,17]. The piRNA expression profile is tissue-specific suggesting that it has functional roles [18]. Additionally, genomic studies have revealed that piRNA expression is deregulated in several diseases, including cancer [19,20,21].

Recently, some studies have investigated genetic variations in piRNAs and have suggested that these polymorphisms may affect the risk of susceptibility to various types of cancer [19,22,23,24]. In this sense, it is noted that changes in genes encoding piRNAs can significantly impact their synthesis and functions.

The importance of piRNA as a regulatory molecule has been previously described. Thus, in this study, we used genomic data from the 1000 Genomes Project [25] and piRBase [26] to analyze its conservation by analyzing single-nucleotide polymorphism (SNP) and insertion/deletion (INDEL) variation patterns, as approached by Bhattacharya and Cui [27] when analyzing miRNAs.

## 2. Materials and Methods

### 2.1. Data Acquisition

The genomic positions of the piRNAs were obtained from the annotation available in piRBase [26], the largest existing database on piRNA containing more than 77 million sequences [26].

The data were obtained from piRBase and the 1000 Genomes Project based on the same reference genome (GRCh37). The piRNA annotation file was extracted in BED format. BEDtools [28] was used to merge piRNAs colocalized in the annotation [29]. piRNAs were divided into two distinct groups: (i) low-repetition piRNAs (~3%)—those with three or fewer repetitions in the human genome; (ii) high repetition piRNAs (~97%)—those with more than three repetitions in the genome. In total, 600,960 piRNA genome positions were investigated.

### 2.2. Statistical Analysis

Data analysis of polymorphisms in piRNAs and conservation graphs of the piRNA regions were obtained with the statistical analysis software R [30]. The flanking and adjacent regions have the same piRNA length: flanking regions are located immediately alongside the 5′ and 3′ piRNA extremities, and adjacent regions are located 1000 bases away in both the 5′ and the 3′ directions. Variant SNPs and INDEL types were obtained from 2504 individuals sequenced by the 1000 Genomes Project [25]. To better understand piRNA region conservation within the human genome, we compared the variation rate of piRNA regions against different genomic regions, such as miRNA, exonic, and intronic/intergenic (non-exonic) regions. Lastly, our analysis included the study of variations among the piRNAs′ nucleotide sequences.

The Kruskal–Wallis test followed by Dunn′s test for multiple comparisons was used to compare variation rates among piRNAs and other genomic regions, such as miRNAs, as well as exonic and non-exonic regions, in addition to piRNAs′ adjacent and flanking regions (the value n for each region is 24 due to the number of chromosomes); this method was also used to compare variation rates among chromosomes on each piRNA′s nucleotide position (the value n for each position is the number of piRNAs, which varies from 821,929 to 368,387 due to their differences in size). This specific statistical approach is suitable because more than three variables are compared in our analysis and because nonparametric tests provide reliable results even when the data and samples do not follow the assumptions of normality.

The libraries vcfR [31] and VariantAnnotation [32] were used in the R platform to quantify variations in piRNA nucleotides. Similar methods and R libraries were applied to quantify the miRNAs and the exonic and non-exonic variations.

## 3. Results

We identified 583,680 variants among 2504 samples from the 1000 Genomes Project in 360,202 piRNA locations (59.94% of piRNAs), of which 98.59% (575,447) were SNPs and 1.41% (8,233) were INDELs. Approximately 40% of the investigated piRNAs did not have any variants in all samples.

Upon analyzing piRNA region variation frequency by chromosome, there was no significant difference among them (95% confidence), except for the sex chromosomes. On the X chromosome, 51.77% of the piRNA regions had at least one SNP, while only 5.54% on the Y chromosome harbored genetic variations. Similar results were obtained for INDELs; the X chromosome presented a rate of 1.92%, and no such variant was found on the Y chromosome.

**Figure 1:**
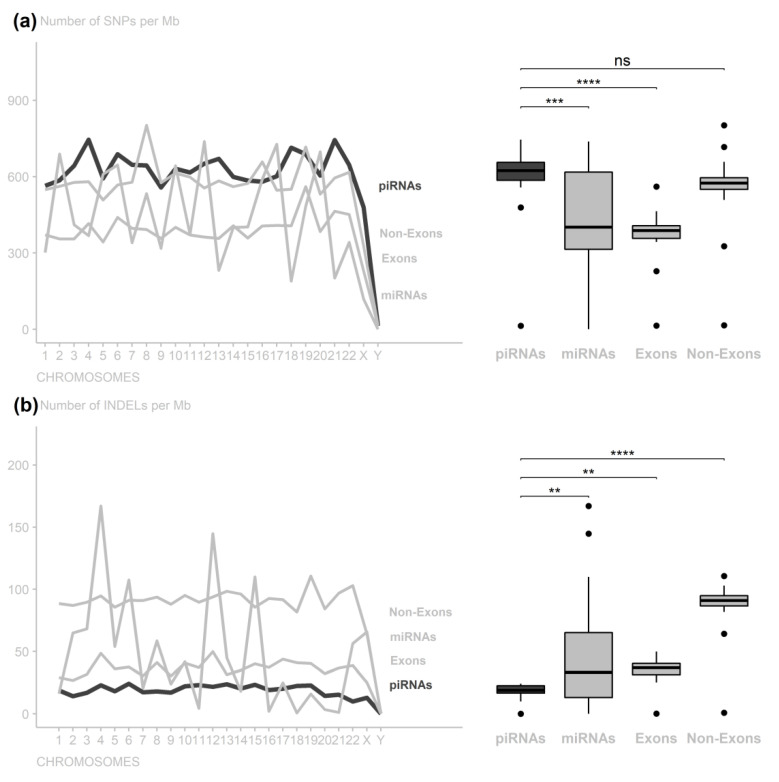
Number of variants per 1,000,000 nucleotides along the chromosomes per genomic region. (**a**) piRNA regions have low conservation compared to exonic and miRNA regions, despite greater miRNA variance, without a significant difference from non-exonic regions. (**b**) piRNA regions are as conserved as exonic regions, with conservation level close to that of miRNA regions (ns: nonsignificant; ** *p*-value < 0.01; *** *p*-value < 0.001; **** *p*−value < 0.0001).

In order to analyze the conservation of piRNA regions, we compared it with other regions with better known conservation degrees (miRNAs, exonic, and non-exonic). When comparing SNP rates in piRNA with different genomic regions, there were significant differences in comparison to miRNAs (adjusted *p*-value = 2.054999 × 10^4^) and exonic (adjusted *p*-value = 1.790354 × 10^7^) but not in those from non-exonic regions (adjusted *p*-value = 5.005757 × 10^1^) (Figure 1a and Supplementary Tables S1 and S2).

According to the INDEL rate, the piRNA regions significantly differed from the miRNA regions (adjusted *p*-value = 1.986334 × 10^2^) but did not differ from the exonic regions (adjusted *p*-value = 2.174223 × 10^1^). The exon INDEL rates also did not differ from the miRNA regions (adjusted *p*-value = 2.174223 × 10^1^). Additionally, the piRNA, miRNA, and exonic rates differed from those in the non-exonic regions (adjusted *p*-values = 1.786511 × 10^4^, 9.627623 × 10^10^, and 7.760338 × 10^7^, respectively) (Figure 1b and Supplementary Tables S3 and S4).

**Figure 2:**
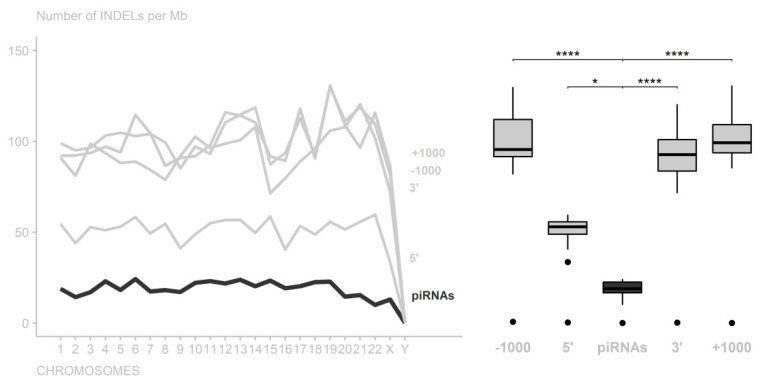
Number of INDELs per 1,000,000 nucleotides in the piRNA regions among their flanking and adjacent regions. In all chromosomes, the piRNAs had the highest conservation level (* *p*-value < 0.05; **** *p*-value < 0.0001).

We also compared piRNA region variations with their flanking (5′ and 3′ extremities) and adjacent regions (±1000 nt), and it was possible to notice for INDELs that piRNA regions presented significant differences compared to the 5′ flanking regions (adjusted *p*-value = 3.897142 × 10^2^), 3′ flanking regions (adjusted *p*-value = 1.120359 × 10^8^), and adjacent regions (−1000 nt with adjusted *p*-value = 3.569849 × 10^11^ and +1000 nt with adjusted *p*-value = 2.272380 × 10^11^) (Figure 2 and Supplementary Tables S5 and S6).

**Figure 3:**
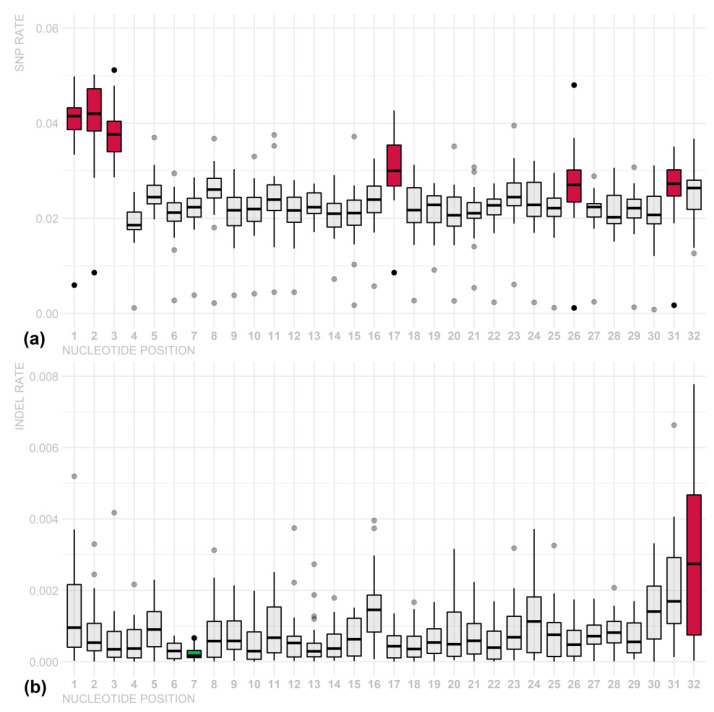
SNP (**a**) and INDEL (**b**) variation rates in piRNA regions per chromosome and nucleotide. Less conserved nucleotides are shown in red, and more conserved nucleotides are shown in blue. Although there were significant differences among some nucleotides (*p*-value < 0.05), this result suggests that there is no specific region of piRNA that stands out as a seed region.

**Figure 4:**
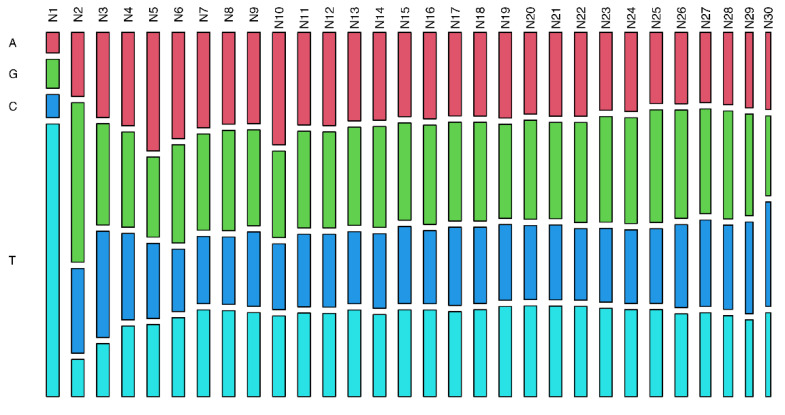
Frequency of each nucleotide base along with piRNA transcripts. The analyzed distribution of the nucleotides by position showed a preference for base U in the first position (79%), G in the second position (46%), and base A in the 10th position (33%).

To verify the presence of a seed region in the piRNA sequences, we analyzed variation rates in piRNAs per chromosome and nucleotide position, and it was not possible to identify outstanding regions, only isolated nucleotide positions. For SNP variants, position 4 was the most conserved, whereas positions 1, 2, and 7 were less conserved. For INDEL polymorphisms, otherwise, no position was highlighted as more conserved; however, positions 1, 31, and 32 presented higher variation rates (Figure 3 and Supplementary Tables S7 and S8). These INDEL variations may be biases due to cloning artefacts and/or non-templated nucleotides at these positions. Additionally, the variation in piRNA size may affect the mutation rates of the last few nucleotides. These results suggest that there is no specific region of piRNA that stands out as a seed region. However, there were nucleotide preferences: U in the first position (79%), G in the second (46%), and A in the 10th (33%) (Figure 4 and Supplementary Tables S7 and S8).

## 4. Discussion

It was observed that the Y chromosome presents different behaviors than the other chromosomes in several aspects due to its unique properties, which involve male specificity, haploidy, and structure predominantly averse to the crossing over phenomenon [33]. There are two types of regions on the Y chromosome with different conservation behaviors: the male-specific region of the Y (MSY) and the pseudoautosomal regions (PAR1 and PAR2) [33,34]. MSY has high variant rates compared to other autosomal chromosomes; however, the PAR1 and PAR2 regions have a very low density of SNPs and almost no INDEL variation since they cause male sterility [35,36]. Therefore, the low rates of SNPs and especially INDEL variants in Y chromosome piRNAs indicate that these changes may result in critical functional changes in cell physiology.

Investigating all of the other chromosome SNP variation rates and comparing them among different genomic structures, the piRNA regions were found to be less conserved in both the exonic and the miRNA regions, and the non-exonic regions did not present different conservation levels when compared to the piRNA regions. Upon observing the INDEL variations, however, no significant differences were observed when compared to the exonic region, and the piRNA regions suggested less conserved structures than the miRNAs and more conserved structures than the non-exonic regions.

The divergent behavior of SNP and INDEL variation rates in the piRNA regions suggested that it has a structure that is more permissible for SNP-type polymorphisms, indicating that these variants have little influence on the function of these structures. For INDEL polymorphisms, however, piRNA regions have a low variation rate, similar to the miRNA regions rate values. The effect of INDELs on miRNAs has been proven to have a significant impact [27], and, similar to piRNAs, these data also indicate a potentially harmful behavior on their functional role.

Concerning INDEL variants, in general, piRNAs, miRNAs, and exonic regions are more conserved, tending to preserve the original structure to avoid loss or deregulation of their function. These observations may suggest that there is selective pressure against genetic variations in piRNAs, mainly because INDEL polymorphisms possibly have more impact than SNPs [37,38] since substitution of a base probably does not interfere with the regulatory function of piRNA, as pairing with the target region does not need to be perfect [12].

The piRNA nucleotide structure is characterized by one U at the 5′ end, and this nucleotide is needed for PIWI recognition protein and endonucleolytic cleavage by Zucchini protein (which acts in piRNA processing) [39,40]. In addition, studies have demonstrated that three proteins can bind to the piRNA: PIWI, AUB, and AGO3; PIWI is more frequently involved, and AUB and AGO3 bind less often. PIWI- and AUB-bound RNAs have a strong preference for a 5′ end uridine, a trend that is not present in AGO3-bound piRNAs. AUB pairs to its target mRNA and induces cleavage, generating piRNA with A at position 10 that is recognized by AGO3, since this piRNA that binds to AGO3 has enrichment for A, the complement of the 5′ U, at position 1 present in piRNAs that bind to AUB [39,40,41,42].

Additionally, the G base in the second position was also identified by Gebert and Ketting [43], which suggests a conserved role of the Piwi/piRNA pathway in posttranscriptional regulation in mammals. These findings agree with our results; the distribution of nucleotides showed a preference of base U in the first position, base A in the 10th position, and base G in the second position.

One of the regulatory mechanisms described for piRNA is posttranscriptional silencing by pairing in the 3′UTR of mRNAs, similar to the miRNA acting model [16]. Saunders and colleagues [44] were the first to observe a low-level gene variation in miRNAs, especially mature miRNAs and their seed regions, compared to surrounding regions. In miRNAs, the seed region is predominantly defined as falling between the second and eighth bases, counted from the 5′ end of the structure [45].

Thus, to investigate the rules adjacent to target piRNA binding, specific and nonspecific sequence-based functions have been proposed. Zhang and colleagues [46], researching the role of piRNAs in *Caenorhabditis elegans*, found results that reinforce specific sequence binding, giving a region between the second and seventh nucleotides a critical role in pairing with the target region. This is precisely the role of the seed in miRNAs, showing a possible similarity in both structures′ target-specific mechanisms. However, it should be noted that the non-seed region would also be necessary for the recognition function, although it is more permissive to alterations. The non-seed region allows for some variations (at most three) to occur with no interference with its function, whereas the seed region is not permissive, completely misrepresenting the recognition function if a single variation occurs in this region.

Analyzing Figure 3, we observed that there is no region in the piRNA sequences that can be considered a seed sequence, including the one defined by Zhang and colleagues [46]. This may indicate that all the piRNA sequence is equally essential for interacting with their target sites. Our findings could not identify any more conserved position; however, it showed some particular nucleotides with significantly higher INDEL variation rates at positions 1, 31, and 32, which can be explained by the tolerance of a few modifications of piRNA sequences already described in the literature [46,47].

Several studies suggest a seed region in piRNAs, although there is little consensus on this matter. Rojas-Ríos and Simonelig [48] proposed that it needs a perfect match at nucleotides 2–11 and fewer than five mismatches at nucleotides 12–21, whereas Shen and colleagues [47], studying *C. elegans,* proposed that the seed sequence (i.e., positions 2–8) and supplemental nucleotides near the 3′ end (positions 14–19) of the piRNA are important determinants of piRNA target binding and silencing. In addition, our results corroborate the findings of Vourekas and colleagues [49]; base-paired piRNAs in *Drosophila melanogaster* revealed a preference to utilize nucleotides at positions 2–6 with additional base pairs at positions 16–24, and this suggests that piRNAs do not utilize a conserved seed sequence, although the mechanics of piRNA complementary binding are analogous to those of microRNAs.

The performed analysis has some possible biases since the 1000 Genomes Project data have low coverage in intronic and intergenic regions, which may alter some mutation rates identified in the piRNA regions; nevertheless, we believe that the sample size (2504 samples) is large enough to minimize this bias. Another limitation is that we inferred conservation on the basis of mutation rates rather than comparing species. However, we believe that our approach allows robust results since INDEL- and SNP-type polymorphisms in all regions of piRNAs were investigated and compared with all other genomic regions.

Analyzing the conservation of piRNA regions considering the polymorphism rate and the presence of a seed region in mature piRNA sequence allowed us to infer the impact of variants on the piRNA sequence. This allows directing analyses that investigate how the pairing of piRNAs to the transcript occurs since it may not have a specific pairing region, thus recognizing different transcripts and amplifying their functional effect on epigenetic regulation. Furthermore, studies like this help elucidate issues related to the structure of piRNAs and their genomic region, contributing to understanding their biology and function.

## 5. Conclusions

In general, it was observed that piRNA regions have higher conservation for INDEL variants and lower conservation for SNP variants relative to exonic regions. This suggests that piRNA regions are more permissive to SNP variations and less permissive to INDEL variations, indicating that SNPs may have little effect on piRNA regular activity, and that INDELs may have a significant impact on its structure and functions. Analysis of the surrounding regions indicated that piRNA regions were more conserved than the flanking and adjacent regions (±1000 nt). In addition, in this study, the Y chromosome presented unique conservation patterns as compared to the other chromosomes.

Lastly, our analyses suggest that there is no specific region of piRNA that can be considered a seed, as occurs in miRNAs, since the conservation degree of piRNAs as a whole did not allow for highlighting a more conserved region or a specific genomic position. This may imply that the entire structure of piRNAs, allowing for a few modifications, is important for them to carry out their roles in regulating gene expression. However, further studies are needed to examine the effects of variants on piRNA function.

## Data Availability

All data analyzed were obtained from piRBase and the 1000 Genomes Project based on the same reference genome (GRCh37). Annotations available in piRBase were used to describe genomic positions of the piRNAs. The piRNA annotation file was extracted in BED format. BEDtools was used to merge piRNAs colocalized in the annotation. Variant SNPs and INDEL types were obtained from 2504 individuals sequenced by the 1000 Genomes Project.

## Supplementary Materials

The following are available online at https://www.mdpi.com/article/10.3390/cimb44020062/s1.
