# Supplementary material for: Identification and Characterization of Polymorphisms in piRNA Regions
Source: Curr Issues Mol Biol. 2022 Feb 15;44(2):942–51. doi: 10.3390/cimb44020062 (PMC8929088; doi:10.3390/cimb44020062)
Supplement: Supplementary file 1 [file cimb-44-00062-s001.zip › cimb-1522006-supplementary.pdf]

Supplementary Materials:

Supplementary Table S1. SNP rates per chromosome and genomic structure.

Chrom	Type	piRNAs	miRNAs	Exons	Non-Exons
1	SNP	5.643e-04	3.016e-04	3.715e-04	5.487e-04
2	SNP	5.859e-04	6.888e-04	3.553e-04	5.624e-04
3	SNP	6.426e-04	4.112e-04	3.551e-04	5.775e-04
4	SNP	7.453e-04	3.679e-04	4.156e-04	5.804e-04
5	SNP	5.933e-04	6.097e-04	3.433e-04	5.084e-04
6	SNP	6.883e-04	6.458e-04	4.397e-04	5.673e-04
7	SNP	6.467e-04	3.408e-04	3.971e-04	5.782e-04
8	SNP	6.440e-04	5.335e-04	3.921e-04	8.016e-04
9	SNP	5.576e-04	3.182e-04	3.571e-04	5.764e-04
10	SNP	6.310e-04	6.429e-04	4.010e-04	6.141e-04
11	SNP	6.166e-04	3.726e-04	3.710e-04	5.986e-04
12	SNP	6.513e-04	7.381e-04	3.628e-04	5.554e-04
13	SNP	6.702e-04	2.320e-04	3.577e-04	5.839e-04
14	SNP	5.988e-04	4.005e-04	4.068e-04	5.608e-04
15	SNP	5.847e-04	4.022e-04	3.587e-04	5.734e-04
16	SNP	5.802e-04	5.899e-04	4.065e-04	6.580e-04
17	SNP	6.017e-04	7.270e-04	4.082e-04	5.466e-04
18	SNP	7.138e-04	1.897e-04	4.068e-04	5.506e-04
19	SNP	6.887e-04	4.832e-04	5.610e-04	7.169e-04
20	SNP	6.044e-04	6.978e-04	3.840e-04	5.313e-04
21	SNP	7.440e-04	2.017e-04	4.639e-04	5.951e-04
22	SNP	6.459e-04	3.420e-04	4.514e-04	6.170e-04
X	SNP	4.787e-04	1.182e-04	2.287e-04	3.264e-04
Y	SNP	1.350e-05	0.000e+00	1.432e-05	1.527e-05

Supplementary Table S2. Pairwise comparisons of INDEL rates in piRNA, miRNA, exonic and non-exonic regions.

Group 1	Group 2	p-value adjusted	p-value adjusted significant
piRNAs	miRNAs	3.518e-04	***
piRNAs	Exons	2.116e-07	****
piRNAs	Non-Exons	8.922e-02	ns
miRNAs	Exons	8.922e-02	ns
miRNAs	Non-Exons	6.028e-02	ns
Exons	Non-Exons	3.518e-04	***

Dunn's-test for multiple comparisons of independent samples and p-values adjusted by Benjamini-Hochberg's method.

Supplementary Table S3. INDEL rates per chromosome and genomic structure.

Chrom	Type	piRNAs	miRNAs	Exons	Non-Exons
1	INDEL	1.885e-05	1.619e-05	2.909e-05	8.857e-05
2	INDEL	1.424e-05	6.495e-05	2.687e-05	8.699e-05
3	INDEL	1.698e-05	6.817e-05	3.171e-05	8.955e-05
4	INDEL	2.301e-05	1.670e-04	4.857e-05	9.467e-05
5	INDEL	1.822e-05	5.400e-05	3.599e-05	8.559e-05
6	INDEL	2.417e-05	1.075e-04	3.751e-05	9.108e-05
7	INDEL	1.738e-05	2.067e-05	3.054e-05	9.094e-05
8	INDEL	1.806e-05	5.851e-05	4.110e-05	9.376e-05
9	INDEL	1.704e-05	2.400e-05	3.036e-05	8.794e-05
10	INDEL	2.215e-05	4.178e-05	4.069e-05	9.512e-05
11	INDEL	2.306e-05	4.439e-06	3.716e-05	8.955e-05
12	INDEL	2.176e-05	1.447e-04	4.986e-05	9.393e-05
13	INDEL	2.380e-05	4.502e-05	3.154e-05	9.839e-05
14	INDEL	2.030e-05	1.822e-05	3.499e-05	9.615e-05
15	INDEL	2.335e-05	1.099e-04	4.012e-05	8.564e-05
16	INDEL	1.909e-05	2.120e-06	3.722e-05	9.262e-05
17	INDEL	2.023e-05	2.484e-05	4.390e-05	9.167e-05
18	INDEL	2.246e-05	7.073e-07	4.094e-05	8.173e-05
19	INDEL	2.277e-05	1.589e-05	4.040e-05	1.106e-04
20	INDEL	1.452e-05	3.408e-06	3.227e-05	8.429e-05
21	INDEL	1.542e-05	1.099e-06	3.672e-05	9.691e-05
22	INDEL	9.950e-06	5.640e-05	3.878e-05	1.029e-04
X	INDEL	1.290e-05	6.570e-05	2.521e-05	6.413e-05
Y	INDEL	0.000e+00	0.000e+00	1.929e-07	8.731e-07

Supplementary Table S4. Pairwise comparisons of INDEL rates in piRNA, miRNA, exonic and non-exonic regions.

Group 1	Group 2	p-value adjusted	p-value adjusted significant
-1000	5'	5.839e-06	****
-1000	piRNAs	2.912e-11	****
-1000	3'	3.077e-01	ns
-1000	+1000	8.259e-01	ns
5'	piRNAs	3.897e-02	*
5'	3'	5.527e-04	***
5'	+1000	2.441e-06	****
piRNAs	3'	2.254e-08	****
piRNAs	+1000	1.214e-11	****
3'	+1000	2.390e-01	ns

Dunn's-test for multiple comparisons of independent samples and p-values adjusted by Benjamini-Hochberg's method.

Supplementary Table S5. INDEL rates in piRNA, flanking and adjacent regions.

Chrom	Type	-1000	5'	piRNAs	3'	+1000
chr1	INDEL	9.205e-05	5.461e-05	1.885e-05	9.092e-05	9.893e-05
chr2	INDEL	9.222e-05	4.393e-05	1.424e-05	8.117e-05	9.506e-05
chr3	INDEL	9.359e-05	5.279e-05	1.698e-05	9.861e-05	9.647e-05
chr4	INDEL	9.706e-05	5.104e-05	2.301e-05	9.353e-05	1.032e-04
chr5	INDEL	9.399e-05	5.302e-05	1.822e-05	8.813e-05	1.047e-04
chr6	INDEL	1.145e-04	5.829e-05	2.417e-05	8.883e-05	1.029e-04
chr7	INDEL	1.045e-04	4.928e-05	1.738e-05	8.438e-05	1.041e-04
chr8	INDEL	8.657e-05	5.466e-05	1.806e-05	7.885e-05	9.947e-05
chr9	INDEL	9.092e-05	4.113e-05	1.704e-05	9.181e-05	8.509e-05
chr10	INDEL	9.184e-05	4.882e-05	2.215e-05	1.025e-04	9.719e-05
chr11	INDEL	9.804e-05	5.494e-05	2.306e-05	9.637e-05	9.316e-05
chr12	INDEL	1.159e-04	5.666e-05	2.176e-05	9.877e-05	1.104e-04
chr13	INDEL	1.143e-04	5.670e-05	2.380e-05	1.006e-04	1.147e-04
chr14	INDEL	1.104e-04	4.953e-05	2.030e-05	1.079e-04	1.186e-04
chr15	INDEL	9.175e-05	5.859e-05	2.335e-05	7.138e-05	8.737e-05
chr16	INDEL	8.921e-05	4.041e-05	1.909e-05	7.978e-05	9.375e-05
chr17	INDEL	1.180e-04	5.343e-05	2.023e-05	8.881e-05	1.127e-04
chr18	INDEL	9.056e-05	4.875e-05	2.246e-05	9.615e-05	9.224e-05
chr19	INDEL	1.299e-04	5.562e-05	2.277e-05	1.059e-04	1.307e-04
chr20	INDEL	1.112e-04	5.155e-05	1.452e-05	1.078e-04	1.087e-04
chr21	INDEL	1.188e-04	5.567e-05	1.542e-05	1.204e-04	9.656e-05
chr22	INDEL	1.094e-04	5.957e-05	9.950e-06	1.018e-04	1.157e-04
chrX	INDEL	8.168e-05	3.353e-05	1.290e-05	7.219e-05	8.648e-05
chrY	INDEL	6.633e-07	3.078e-07	0.000e+00	2.487e-07	2.956e-08

Supplementary Table S6. Pairwise comparisons of INDEL rates in piRNA, flanking and adjacent regions.

Group 1	Group 2	p-value adjusted	p-value adjusted significant
-1000	5'	5.839e-06	****
-1000	piRNAs	2.912e-11	****
-1000	3'	3.077e-01	ns
-1000	+1000	8.259e-01	ns
5'	piRNAs	3.897e-02	*
5'	3'	5.527e-04	***
5'	+1000	2.441e-06	****
piRNAs	3'	2.254e-08	****
piRNAs	+1000	1.214e-11	****
3'	+1000	2.390e-01	ns

Dunn's-test for multiple comparisons of independent samples and p-values adjusted by Benjamini-Hochberg's method.

Supplementary Table S7. SNP rates in piRNA regions by nucleotide (NT).

Chrom	Type	NT 1	NT 2	NT 3	NT 17	NT 26	NT 31
chr1	SNP	3.819e-02	4.141e-02	3.165e-02	3.223e-02	2.514e-02	2.874e-02
chr2	SNP	4.329e-02	4.040e-02	3.447e-02	2.855e-02	2.504e-02	2.357e-02
chr3	SNP	4.075e-02	4.718e-02	3.572e-02	2.796e-02	2.112e-02	3.022e-02
chr4	SNP	3.923e-02	5.026e-02	4.246e-02	3.535e-02	2.739e-02	3.201e-02
chr5	SNP	4.170e-02	4.007e-02	3.421e-02	2.933e-02	2.800e-02	2.682e-02
chr6	SNP	4.257e-02	3.681e-02	4.334e-02	3.136e-02	2.346e-02	2.705e-02
chr7	SNP	4.551e-02	4.324e-02	4.012e-02	3.558e-02	2.570e-02	3.242e-02
chr8	SNP	4.240e-02	2.850e-02	3.336e-02	3.176e-02	3.077e-02	2.667e-02
chr9	SNP	4.132e-02	4.248e-02	3.089e-02	2.972e-02	2.008e-02	3.256e-02
chr10	SNP	4.177e-02	4.151e-02	4.513e-02	3.659e-02	2.303e-02	2.473e-02
chr11	SNP	3.991e-02	4.916e-02	3.765e-02	3.121e-02	3.203e-02	2.749e-02
chr12	SNP	4.259e-02	3.859e-02	3.549e-02	4.037e-02	2.733e-02	3.020e-02
chr13	SNP	4.503e-02	4.744e-02	3.606e-02	4.267e-02	2.826e-02	3.016e-02
chr14	SNP	4.982e-02	3.770e-02	2.864e-02	2.384e-02	2.301e-02	2.833e-02
chr15	SNP	3.414e-02	4.556e-02	3.840e-02	2.458e-02	2.683e-02	2.048e-02
chr16	SNP	4.357e-02	5.000e-02	3.051e-02	2.724e-02	3.064e-02	2.592e-02
chr17	SNP	3.360e-02	4.060e-02	3.784e-02	2.516e-02	3.692e-02	2.520e-02
chr18	SNP	4.743e-02	4.678e-02	3.937e-02	2.380e-02	2.498e-02	3.175e-02
chr19	SNP	4.324e-02	4.874e-02	4.127e-02	2.676e-02	3.011e-02	3.507e-02
chr20	SNP	3.883e-02	3.652e-02	4.787e-02	3.032e-02	3.030e-02	2.175e-02
chr21	SNP	3.883e-02	4.982e-02	5.119e-02	3.640e-02	4.802e-02	2.828e-02
chr22	SNP	3.340e-02	4.444e-02	3.904e-02	3.597e-02	2.337e-02	1.897e-02
chrX	SNP	3.636e-02	3.608e-02	2.929e-02	2.682e-02	2.958e-02	2.456e-02
chrY	SNP	5.970e-03	8.600e-03	3.770e-02	8.600e-03	1.160e-03	1.720e-03

Only NTs with highest or lowest conservations.

Supplementary Table S8. INDEL rates in piRNA regions by nucleotide (NT).

Chrom	Type	NT 7	NT 32
chr1	INDEL	1.000e-05	1.850e-03
chr2	INDEL	1.500e-04	5.530e-03
chr3	INDEL	4.000e-05	2.110e-03
chr4	INDEL	2.400e-04	2.200e-04
chr5	INDEL	6.000e-05	6.000e-05
chr6	INDEL	3.100e-04	1.680e-03
chr7	INDEL	1.700e-04	3.030e-03
chr8	INDEL	2.300e-04	8.400e-04
chr9	INDEL	1.400e-04	7.780e-03
chr10	INDEL	5.100e-04	1.720e-03
chr11	INDEL	1.300e-04	4.960e-03
chr12	INDEL	3.800e-04	4.740e-03
chr13	INDEL	2.300e-04	3.000e-05
chr14	INDEL	5.900e-04	3.000e-05
chr15	INDEL	1.000e-05	3.450e-03
chr16	INDEL	6.700e-04	4.910e-03
chr17	INDEL	3.300e-04	3.210e-03
chr18	INDEL	2.200e-04	2.740e-03
chr19	INDEL	1.400e-04	3.270e-03
chr20	INDEL	1.100e-04	4.000e-05
chr21	INDEL	1.200e-04	6.280e-03
chr22	INDEL	3.300e-04	6.600e-04
chrX	INDEL	6.000e-05	4.610e-03
chrY	INDEL	NA	NA

Only NTs with highest or lowest conservations.
